# Ordered sacrifice for survival: insights from drought-stressed fine roots in soybean

**DOI:** 10.1093/plphys/kiag152

**Published:** 2026-03-16

**Authors:** Guannan Wang

**Affiliations:** Assistant Features Editor, Plant Physiology, American Society of Plant Biologists, Rockville, MD, United States; Department of Biology, Stanford University, Stanford, CA, United States; Howard Hughes Medical Institute, Department of Biology, Stanford University, Stanford, CA, United States

Fine roots, historically defined as roots that are ≤ 2 mm in diameter, are responsible for acquiring essential soil resources for plant growth and development, interacting with surrounding soil environments, and mediating biogeochemical cycling in terrestrial ecosystems (McCormack et al. 2015). While they account for as little as 2% of the root network in woody species, fine roots generally make up most, if not all, of the entire root system in herbaceous plants, which include over two-thirds of all crops (Meyer et al. 2012). Throughout the plant lifecycle, fine roots continually emerge, age, and die in a process that represents a major consumer of global net primary production (Lukac 2012).

Over the past 3 decades, research has substantially advanced our understanding of fine-root responses to environmental challenges, particularly drought. As soil water availability decreases, fine roots experience a rapid and precipitous decline in radial hydraulic conductance, driven by the loss of turgor pressure and noticeable shrinkage of thin-walled cortex cells. As dehydration intensifies, this cellular shrinkage eventually leads to mechanical failure and localized collapse and death of the root cortex. The structural degradation results in the formation of extensive cortex lacunae (air spaces in the cortical tissue), which physically disrupt the radial pathway for water transport and create air gaps that isolate the central vascular system. As a consequence, the plant is deliberately decoupled from the drying soil to prevent water loss and the buildup of hydrostatic tension in the xylem (Cuneo et al. 2016). While the specific declines in conductivity and end-stage tissue collapses are well-documented, the dynamic cell-level hydraulic behaviors within fine root networks—from early dehydration to structural failure and ultimate mortality—remain poorly characterized.

In this issue of *Plant Physiology*, Harrison Day and colleagues (Harrison Day et al. 2026) investigated the spatial physiological changes of fine roots from the drought-sensitive staple crop soybean (*Glycine max*) when exposed to different drought gradients. To examine the impact of water stress on different organs, the authors simultaneously quantified the in situ water potential of leaves, stems, and fine roots from the same soybean plants subjected to intensifying drought conditions (until stem water potential reached −2.0 MPa) using thermocouple psychrometers, which determine tissue's water potential by measuring the vapor pressure of water in equilibrium with the tissue inside a sealed chamber. Fine roots exhibited a declining trend in water potential that is highly similar to that of the leaves and stems until 50% of the whole-plant xylem embolized (P_50_, −1.75 MPa), confirming the hydraulic equilibrium among these organs. The vulnerability of xylem to embolism was closely comparable across leaves, stems, and roots as the water potentials at which 12%, 50%, and 88% of the xylem became embolized (termed as P_12_, P_50_, P_88_, respectively) remained indistinguishable among these organs.

When the severity of drought treatment was extended further to −4.0 MPa, the authors found that fine roots in soybean underwent 3 phases: 1) a rapid decline of roughly 50% in root diameter under extremely mild water stress (stem water potential of −0.10 MPa); 2) continued but slowed shrinkage (from about 55% to 85%) under moderate water stresses (between −0.25 and −2 MPa); and 3) stabilized shrinkage reaching over 90% under severe drought (stem water potential more negative than −3 MPa). Roughly 76% of the root shrinkage occurred prior to the point of incipient embolism in roots (Fig. 1), which refers to the specific water potential threshold at which embolism starts to form stably within the xylem network and is often quantified as water potential at P_12_ (Brodribb et al. 2016). These results suggested that the early fine root shrinkage during drought cannot be attributed to differential xylem tension across the plants.

**Figure 1:**
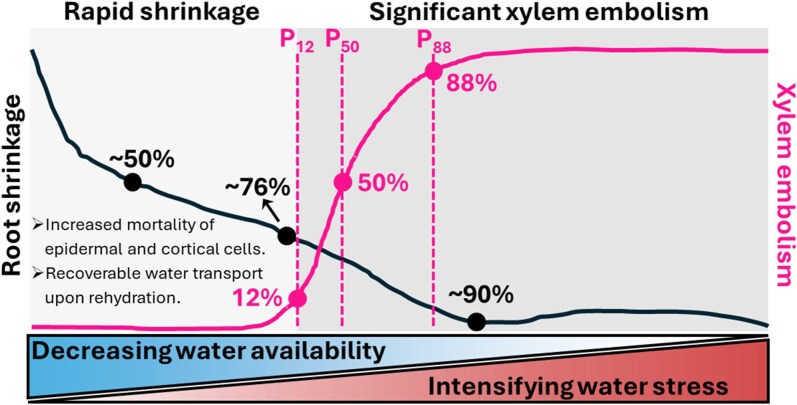
The dynamics of fine root responses to drought in soybean. P_12_, P_50_, and P_88_ refer to the water potentials at which 12%, 50%, and 88% of the xylem became embolized. Source Data from Brodersen et al. 2025.

Next, the authors used fluorescein diacetate to evaluate the viability of cells to identify the tissues that might contribute to the early shrinkage. Fluorescein diacetate is a cell-permeable esterase substrate that can be hydrolyzed by esterases to fluorescent fluorescein, which accumulates only in living cells with intact membranes (Widholm 1972). The death of epidermal cells and outermost cortex cells increased significantly starting at the stem water potential of −0.25 MPa, and these tissues were mostly dead by −1.0 MPa, prior to significant whole plant xylem embolism P_12_ (around −1.35 MPa, Fig. 1). On the contrary, stele cells remained viable until water potential reached −1.75 MPa, which corresponded to the whole plant P_50_.

To directly quantify the turgor changes in root cells when exposed to drought, the authors used a novel noninvasive approach, cavitation bubble manometry (CBM), that recently has been successfully applied to leaf epidermal and guard cells (Brodersen et al. 2025). This method creates nucleated microbubbles in surface cells of intact plants. As the maximum radius of microbubbles is constrained by the pressure of the surrounding liquid, the cavitation microbubble dynamics can therefore serve as a proxy measurement of cell turgor pressure. In the soybean plants, when xylem water potential declined from 0 MPa to −0.25 MPa, the authors observed an increase in microbubble radius within the intact root epidermal cells, which indicated a decline in the turgor pressure of these cells. The turgor pressure of these cells was completely lost by −0.5 MPa. When the xylem water potential in the fine roots was restored from −0.1 to 0 MPa by rehydration, the turgor pressure of epidermal cells was fully recovered to the unstressed state. A similar, but not complete, recovery of cell turgor after rehydration was also observed for epidermal cells in fine roots exposed to −0.5 MPa. However, further tests using completely air-dried roots revealed that this likely represents passive water absorption rather than active cellular recovery, as these dead roots can regain some hydration state but remain at or near atmospheric pressure with no structural integrity to actively repressurize. Consequently, the viability of epidermal or cortical cells was not restored after rehydration in fine roots dehydrated to −0.5 MPa, nor in those dehydrated to −1.0 MPa. Despite the lack of viability restoration in root cells, stem water potential showed an exponential recovery, and plants dehydrated to −0.5 MPa recovered noticeably faster than those from −1.0 MPa. Consistently, the hydraulic conductance of roots rehydrated from −0.5 MPa was 4 times higher than that of those rehydrated from −1.0 MPa. This suggests that as dehydration intensifies to −1.0 MPa, the complete mortality of the cortex and the early embolism of vulnerable root segments create persistent physical damage, significantly increasing hydraulic resistance and slowing the plant's ability to transport water.

Overall, the findings presented by Harrison Day and colleagues (Harrison Day et al. 2026) revealed the physiological dynamics of different cell layers in the soybean roots during water stress. Epidermal and cortical cells, but not stele cells, exhibited rapid viability loss and contributed primarily to the massive early shrinkage of fine roots in soybean. The shrinkage and death of the epidermal and cortical cells slowed down root water uptake but did not entirely prevent the rehydration of the surviving stele and the whole plant. These discoveries raise several intriguing questions for future research. First, the precise hydraulic pathways allowing water to transport across dead epidermal and cortical cells to reach the living stele during recovery remain unresolved. Furthermore, it is critical to determine whether this ordered, differential cell death is a conserved mechanism across diverse plant species, especially in those that are pre-adapted to drought stresses, and how this localized mortality is regulated. Finally, future investigations should examine how roots interact with complex field soil environments and under which environmental contexts this transient decoupling strategy provides the greatest adaptive benefit.

## Recent related articles in *Plant Physiology*:

- Liu et al. (2023) investigated evaporation-driven hydraulic redistribution (EDHR) in Chinese white poplar (*Populus tomentosa*) under drought stress.
- Yang et al. (2023) examined the relationship between soil-root interface hydraulic conductance and responses of photosynthesis to drought in rice and wheat.

## Data Availability

No new data were generated or analyzed in support of this research.
